# Editorial: Virtual and robotic embodiment

**DOI:** 10.3389/fnrgo.2026.1805149

**Published:** 2026-04-01

**Authors:** Giacinto Barresi, Klaus Gramann, Giovanni Vecchiato, Gaetano Tieri, Philipp Beckerle

**Affiliations:** 1 Bristol Robotics Laboratory, School of Engineering, CATE, University of the West of England, Bristol, United Kingdom; 2 Biological Psychology and Neuroergonomics, Technische Universität Berlin, Berlin, Germany; 3 Institute of Neuroscience, National Research Council of Italy, Parma, Italy; 4 Department of Theoretical and Applied Sciences, eCampus Università, Novedrate, Italy; 5 Virtual Reality and Digital Neuroscience Lab, Department of Law and Digital Society, University of Rome Unitelma Sapienza, Rome, Italy; 6 Department of Electrical Engineering, Friedrich-Alexander-Universität Erlangen-Nürnberg, Erlangen, Germany; 7 Department Artificial Intelligence in Biomedical Engineering, Friedrich-Alexander-Universität Erlangen-Nürnberg, Erlangen, Germany

**Keywords:** embodiment, human-computer interaction, illusions, neuroergonomics, presence, prosthetics, robotics, virtual reality

Exploring ways to transfer or expand the users' body representations through artificial (digital and physical) extensions has become a focal point of interest within converging interdisciplinary research fields, ranging from cognitive science and neuroergonomics to computer and robotics engineering. This reflects a growing commitment to understanding and shaping embodiment phenomena (with their multiple interpretations, e.g., making a person perceive an external object as part of their body) in human-technology interactions (Zbinden et al., 2022; Riva, 2008; Chevalier et al., 2015). The pursuit of embodiment research seeks goals like optimizing the user experience and system control through human-centered design activities that are informed by disciplines like cognitive science and human factors. This could be achieved by three cardinal research directions: (i) exploring the neurocognitive processes underlying body ownership and agency as foundational mechanisms for self-representation (Longo et al., 2008), (ii) understanding the virtual and robotic systems that humans embody during mediated interactions, and (iii) examining the interfaces facilitating bidirectional sensorimotor exchange between humans and these artificial extensions.

In the context of this Research Topic, attention was directed specifically toward embodiment assessment across virtual and robotic contexts. The rubber limb illusion experiment represents a central paradigm of empirical embodiment studies (Botvinick and Cohen, 1998). In these studies, participants observe an artificial hand and embody it through multisensory stimulation of the fake and their real limb. The published contributions investigate body perception through virtual hand illusion paradigms under synchronized and asynchronized conditions, the influence of first-person perspective on out-of-body experiences, and EEG-based detection of error-related potentials in brain-computer interfaces. Additional works address avatar embodiment effects on motor imagery training, psychological experiences of shared presence in virtual connected rooms, and frameworks for measuring prosthetic embodiment. These cutting-edge investigations form the nexus of this exploration, advancing transformative approaches to human-centered design through immersive technologies.

Mashiyama et al. investigate how moving multiple hands synchronously and asynchronously influences body perception. Therefore, 20 participants were asked to conduct reaching tasks with a single hand or with nine hands at once in a virtual environment. In an experiment manipulating hand number and movement synchronicity, the authors measured the senses of body ownership and agency on subjective scales. In a second experiment, they measured reactions to threat stimuli considering electrodermal activity and startle response. They conclude that participants could embody multiple hands depending on the attention they put to the avatars.

Aiming at improved embodiment of robotic prostheses, Bliek et al. postulate an interdisciplinary three-staged framework combining measurements and modeling to foster body schema integration of devices. For measurements, they outline the importance of ecologically valid experimental paradigms. Those can help to determine, which spatiotemporal factors modulate the senses of ownership and agency and to create multisensory and cognitive models of their impact on embodiment. Knowing the modulators and having modeled their influence, the authors foresee to optimize robotic prostheses and their human-machine interfaces to foster embodiment.

Harada et al. evaluate the psychological effects of a virtual connected room, a prototype video-meeting system designed to enhance the perception of shared presence by covering an entire wall with a large display. To assess whether this system promotes the “we-mode”—a cognitive state in which team members share task-relevant mental representations—20 participants performed three joint tasks in face-to-face, virtual connected, and standard display-based remote conditions. The tasks included a referential communication task measuring communication efficiency, a joint Simon task assessing co-representation of task rules, and a joint number judgment task evaluating spontaneous perspective-taking. The results show that the virtual connected room produced significant joint Simon effects and facilitated spontaneous perspective-taking, whereas the standard display condition did not. However, communication efficiency did not differ across conditions. These findings suggest that immersive video-meeting environments that enhance the perception of a partner's presence can promote shared cognitive states relevant to collaborative interactions.

Toi et al. investigate how the height of the first-person perspective (1PP) affects the out-of-body experience (OBE) illusion. In their experiment, 15 participants received visuo-tactile stimulation while viewing their own backs through a head-mounted display, with the camera positioned at three different heights: normal (110 cm), short (27 cm), and tall (180 cm). The authors measured OBE illusion intensity through questionnaires assessing body ownership, self-location, and perceived height, along with size and distance estimation tasks. Their results reveal that the OBE illusion occurred at all 1PP heights and was stronger when the perspective was much higher or lower than the typical viewpoint. However, participants' perceived own heights, as well as size and distance estimations of external objects, did not change according to the 1PP height. These findings suggest that the height of the first-person perspective represents an important factor modulating the intensity of bodily illusions, while not affecting the perception of one's own body dimensions or external objects.

Using EEG, Farabbi et al. explore how both the stimulation environment and the likelihood of making an error influence attention and error-related brain responses as well as their classification accuracy. Using a gamified “Racing Mistakes” task, they compare a standard monitor setup with an immersive Virtual Theatre and test two error probabilities (50% and 20%). Their results show that the monitor condition consistently produces clearer and faster ErrP responses, higher levels of attention, and better classification performance than the immersive environment. In addition, less frequent errors (20%) elicit stronger neural responses and greater attentional engagement, leading to classification accuracies above 75%. Overall, the study emphasizes the close relationship between attention and error processing, highlighting the importance of considering both environmental and task design in VR and experiments using EEG for Brain-Computer-Interfaces (BCI).

In a second study using EEG, Vagaja et al. investigate whether inducing a sense of avatar embodiment before motor imagery (MI) training in virtual reality enhances neural markers relevant for motor-imagery BCIs (MI-BCIs). To test this, participants underwent VR-based MI training, with one group receiving a brief embodiment priming phase and a control group without an embodiment phase. While the embodiment manipulation successfully increased the subjective sense of embodiment, no significant differences in neural patterns or offline BCI classification accuracy emerged between groups. These findings suggest that embodiment priming before training does not confer additional neurophysiological benefits beyond those already provided by embodied VR feedback during MI itself, highlighting the importance of timing and context in designing VR-based neurorehabilitation protocols.

In conclusion, this Research Topic provides transformative insights into how digital and physical extensions modulate human body representation. The collected evidence demonstrates that embodiment can extend to multiple virtual limbs through specific attentional allocation (Mashiyama et al.), while the successful integration of robotic prostheses depends on interdisciplinary models that prioritize the spatiotemporal synchronization between human intent and mechanical response (Bliek et al.). Socially, immersive shared environments facilitate “we-mode” cognitive states and spontaneous perspective-taking (Harada et al.). Furthermore, while the height of a first-person perspective acts as a critical modulator for bodily illusion intensity, it does not distort external spatial perception (Toi et al.). However, findings reveal critical boundaries for technological design: traditional displays may still outperform immersive VR in terms of attentional engagement and neural signal detection for brain-computer interfaces (Farabbiet al.). Additionally, the timing of interventions is essential, as explicit embodiment priming prior to training appears neurophysiologically redundant compared to the task-inherent feedback already provided during the motor imagery itself (Vagaja et al.). Collectively, these studies underscore the necessity of interdisciplinary frameworks integrating subjective and objective metrics to optimize the bidirectional sensorimotor exchange between humans and artificial extensions. Ultimately, characterizing the neurocognitive modulators of embodiment will be crucial for advancing neurorehabilitation and human-centered design through emerging immersive technologies.

## References

[B1] Botvinick M. Cohen J. (1998). Rubber hands 'feel'touch that eyes see. Nature 391, 756–756. doi: 10.1038/35784 9486643

[B2] Chevalier P. Martin J.-C. Isableu B. Tapus A. (2015). “Impact of personality on the recognition of emotion expressed via human, virtual, and robotic embodiments,” in 2015 24th IEEE International Symposium on Robot and Human Interactive Communication (Ro-Man) (Kobe: IEEE), 229–234. doi: 10.1109/ROMAN.2015.7333686

[B3] Longo M. R. Schüür F. Kammers M. P. Tsakiris M. Haggard P. (2008). What is embodiment? A psychometric approach. Cognition 107, 978–998. doi: 10.1016/j.cognition.2007.12.004 18262508

[B4] Riva G. (2008). From virtual to real body: virtual reality as embodied technology. J. Cyber Ther. Rehabil. 1, 7–22. doi: 10.1089/cyber.2017.29099.gri

[B5] Zbinden J. Lendaro E. Ortiz-Catalan M. (2022). Prosthetic embodiment: systematic review on definitions, measures, and experimental paradigms. J. Neuro Eng. Rehabil. 19:37. doi: 10.1186/s12984-022-01006-6 35346251 PMC8962549

